# A Complete Whole-Genome Sequence of Ignavigranum ruoffiae Strain CPL 242382-20, an Opportunistic Human Pathogen Recovered from a Breast Cyst

**DOI:** 10.1128/mra.00521-22

**Published:** 2022-12-06

**Authors:** Danae M. Suchan, Kara D. Loos, Keith D. MacKenzie, Sean D. Workman, Lori Johnson, Andrew D. S. Cameron, David C. Alexander

**Affiliations:** a Institute for Microbial Systems and Society, Faculty of Science, University of Regina, Regina, Saskatchewan, Canada; b Department of Biology, Faculty of Science, University of Regina, Regina, Saskatchewan, Canada; c Roy Romanow Provincial Laboratory, Saskatchewan Health Authority, Regina, Saskatchewan, Canada; d Cadham Provincial Laboratory, Diagnostic Services, Shared Health, Winnipeg, Manitoba, Canada; e Department of Medical Microbiology and Infectious Diseases, Rady Faculty of Health Sciences, University of Manitoba, Winnipeg, Manitoba, Canada; Loyola University Chicago

## Abstract

Ignavigranum ruoffiae is a rare human pathogen. Strain CPL 242382-20 was isolated in Manitoba, Canada, from a breast cyst. Whole-genome sequencing was performed with the Oxford Nanopore Technologies MinION and Illumina MiSeq platforms. The circular chromosome is 1,949,382 bp with 39.68% G+C content and 1,765 protein-coding genes.

## ANNOUNCEMENT

Ignavigranum ruoffiae is a rare opportunistic pathogen and the only species of its genus. The initial description of I. ruoffiae was based on analysis of two clinical isolates, including a wound isolate that was designated the type strain (1607-97, CCUG 3765^T^, DSM 15695^T^) (1). Subsequent descriptions of I. ruoffiae are limited to case reports of isolates from skin abscesses (2, 3). Here, we describe Ignavigranum ruoffiae strain CPL 242382-20, an isolate from an infected breast cyst of a 71-year-old woman.

The isolate was submitted to Cadham Provincial Laboratory (Winnipeg, Canada) as a pure culture of Gram-positive, catalase-negative cocci. The organism could not be identified by matrix-assisted laser desorption ionization–time of flight mass spectrometry (MALDI-TOF MS) (Vitek MS with Knowledge Base v3.2; bioMérieux, Inc.), so sequencing was performed. A partial (772 bp) sequence of the 16S rRNA gene was generated by Sanger sequencing. The 16S rRNA gene was amplified using primers 16SF5, 5′-TGAAGAGTTTGATCMTGGC-3′, and 16SR806, 5′-GGACTACYAGGGTATCTAAT-3′. PCR amplicons were labeled with the BigDye Terminator v3.1 cycle sequencing kit sequenced on a SeqStudio genetic analyzer (Applied BioSystems, ON, Canada). MegaBLAST comparison with the GenBank 16S rRNA sequences database provided a preliminary identification of Ignavigranum ruoffiae (4).

To assist with final taxonomic identification, whole-genome sequencing was performed with the Illumina MiSeq and Oxford Nanopore Technologies (ONT) MinION platforms. Strain CPL 242382-20 was cultured on sheep blood agar and incubated at 35°C in 5% CO_2_ for 72 hours. Genomic DNA was extracted using the MasterPure complete DNA and RNA purification kit (Mandel, ON, Canada). For MinION sequencing, genomic DNA was sheared using a Covaris g-TUBE, following the manufacturer’s specifications to generate fragments ≈8 kb in size. The sequencing library was prepared using the ONT native barcoding expansion kit and ligation sequencing kit. Unless otherwise specified, default parameters were used for all software. Basecalling was performed with Guppy (v5.0.16) using the Super Accuracy basecalling model. MinION sequencing generated 167,178 raw reads with a read length *N*_50_ of 2,075 bp. Reads were demultiplexed and trimmed using porechop (v0.3.2pre). Stringent binning requirements were set using a barcode threshold of 85% with a minimum 15% difference from the second-best match while discarding reads containing middle adapters. NanoQC (v0.9.4) (5) was used evaluate read quality, and an additional 40 bp was trimmed from both read ends to reduce nucleotide frequency noise. Reads aligning to a 5.9-kb Escherichia coli plasmid suspected of contamination prior to library preparation were removed using Minimap2 (v2.24) (6). Filtlong (v0.2.1) was used for quality filtering. The top 95% of reads (based on nucleotide quality scores) were retained, and reads shorter than 1,000 bp were discarded. A total of 86,375 reads with an average length of 2,231 bp and Q score of 15.1 were used for genome assembly. The I. ruoffiae CPL 242382-20 genome was assembled *de novo* with Flye (v2.9) (7) using the –nano–raw setting and was long-read polished with medaka (v1.5.0) using the r941_min_sup_g507 model. The Illumina sequencing library was prepared using the Nextera XT DNA library preparation kit (Illumina, CA, USA). The genome was sequenced to 94× depth of coverage with the Illumina MiSeq system using v2 (300-cycle) paired-end sequencing chemistry. Demultiplexed sequences were evaluated with FastQC (v0.11.8) (8) to inform trimming requirements. fastp (v0.23.2) (9) was used to remove adapters and quality trim reads, removing 15 bp from the start and 2 bp from the end of reads and trimming sequences at an average *Q* score of 30 in a 5 bp sliding window. A total of 750,041 paired reads with lengths from 32 to 130 bp were used to polish the ONT genome assembly with two alternating rounds of Polypolish (v0.4.3) (10) and POLCA from MaSuRCA (v3.4.2) (11). The resulting I. ruoffiae CPL 242382-20 genome is a 1,949,382 bp circularized chromosome with a 39.68% G+C content. The genome was annotated using the NCBI Prokaryotic Genome Annotation Pipeline (v6.1) (12), which predicted 1,765 protein-coding genes, 57 RNA-coding genes, and 25 pseudogenes. FastANI (v1.33) (13) comparison of CPL 242382-20 with I. ruoffiae DSM 15695^T^ generated a whole-genome average nucleotide identity of 97.85%.

The release of deidentified data is permitted under the Personal Health Information Act of Manitoba and did not require institutional review board (IRB) review.

### Data availability.

The partial 16S rRNA gene sequence for strain CPL 242382-20 has been deposited in GenBank under accession number ON994914. The whole-genome sequencing assembly has been deposited as NCBI BioProject PRJNA830926 with GenBank accession number CP096206. Raw sequence read files have been deposited in the NCBI Sequence Read Archive under accession numbers SRR18915762 (ONT) and SRR18907539 (Illumina).
